# Neural encoding and functional interactions underlying pantomimed movements

**DOI:** 10.1007/s00429-021-02332-6

**Published:** 2021-07-10

**Authors:** Giulia Malfatti, Luca Turella

**Affiliations:** Center for Mind/Brain Sciences (CIMeC), University of Trento, Corso Bettini 31, 38068 Rovereto, Italy

**Keywords:** Motor system, Action, Tool, Goal, Pantomime, fMRI, MVPA, Connectivity

## Abstract

Pantomimes are a unique movement category which can convey complex information about our intentions in the absence of any interaction with real objects. Indeed, we can pretend to use the same tool to perform different actions or to achieve the same goal adopting different tools. Nevertheless, how our brain implements pantomimed movements is still poorly understood. In our study, we explored the neural encoding and functional interactions underlying pantomimes adopting multivariate pattern analysis (MVPA) and connectivity analysis of fMRI data. Participants performed pantomimed movements, either grasp-to-move or grasp-to-use, as if they were interacting with two different tools (scissors or axe). These tools share the possibility to achieve the same goal. We adopted MVPA to investigate two levels of representation during the planning and execution of pantomimes: (1) distinguishing different actions performed with the same tool, (2) representing the same final goal irrespective of the adopted tool. We described widespread encoding of action information within regions of the so-called “tool” network. Several nodes of the network—comprising regions within the ventral and the dorsal stream—also represented goal information. The spatial distribution of goal information changed from planning—comprising posterior regions (i.e. parietal and temporal)—to execution—including also anterior regions (i.e. premotor cortex). Moreover, connectivity analysis provided evidence for task-specific bidirectional coupling between the ventral stream and parieto-frontal motor networks. Overall, we showed that pantomimes were characterized by specific patterns of action and goal encoding and by task-dependent cortical interactions.

## Introduction

Pantomimes are a special category of movements. They can convey complex information about our pretended intentions without involving any real interaction with the external environment. While pantomiming a movement without a tool, we can pretend to use the same tool to perform different actions—e.g. grasp scissors to move from one position on the table to a new one or to use them for cutting. At the same time, we can pretend to achieve the same final goal with different tools—e.g. cut an object with scissors, a knife or a scalpel. These examples support the idea that pantomimes can carry information not only about the specific action we pretend to perform, but also about its underlying general goal. Yet, it is still unclear how the brain represents and transfers action and goal information during pantomimed movements. The aim of our study was indeed to better characterize the neural encoding and functional interactions underlying pantomimes which are still poorly understood.

The so-called “tool” network is the likely candidate underlying the neural representations and functional interactions characterizing pantomimed movements (Johnson–Frey 2004; Lewis 2006; Valyear et al. 2017; Buxbaum 2017). This left lateralized cortical network is supported by the functional interactions between regions of the ventral stream—like the posterior middle temporal gyrus (pMTG)—and of the parieto-frontal motor networks—including the supramarginal gyrus (SMG) and the anterior intraparietal sulcus (aIPS) within the inferior parietal lobe (IPL), the superior parietal lobe (SPL), the superior parieto-occipital cortex (SPOC) and the frontal cortex, comprising ventral and dorsal premotor cortices (PMv, PMd).

Neuroimaging studies adopting classical univariate approach showed the consistent recruitment of regions within this network while observing tool images (Chao et al. 1999; Chao and Martin 2000) or while performing a real or pantomimed movement with a tool (Rumiati et al. 2004; Johnson–Frey et al. 2005; Hermsdörfer et al. 2007; Króliczak and Frey 2009; Gallivan et al. 2013c; Brandi et al. 2014; Styrkowiec et al. 2019). However, univariate approach provided only indirect evidence of the possible processing happening within the regions of this network, as it cannot test the informational content represented within a cortical region, which is only possible with MVPA (Kriegeskorte and Bandettini 2007). Moreover, univariate analysis allows to show the recruitment of the tool network in a specific task, but does not allow to understand the functional interplay between regions of this network.

With our study, we aimed at further characterizing the functional contribution and the interactions of the regions within the tool network during pantomimed movements. To this end, we adopted a combination of MVPA and connectivity analysis to investigate: (1) which regions host action and goal information during the planning and execution of pantomimes, (2) the transfer of information between regions of the ventral stream and of parieto-frontal pathways within the network.

To address the first question, we focused on action and goal representations adopting a delayed pantomiming task, which allowed dissociating the two phases of the movement—i.e. planning and execution. We tested action representations with MVPA by comparing patterns of activity for different pantomimes (grasp-to-move vs. grasp-to-use) pretended to be performed with a specific tool (scissors or axe). Then, we investigated the representation of goal information, i.e. the final aim of the pantomime (grasp-to-move vs. grasp-to-use) irrespective of the adopted tool.

We expected to demonstrate widespread encoding of action information within the tool network, as most of its regions represent specific real tool and hand movements (Gallivan et al. 2013c). Regarding goal encoding, the possible description of this type of information might allow to draw inferences on the specific role of premotor, parietal and temporal nodes of the network. We expect to show goal encoding within regions of the IPL (SMG, aIPS), as reported in recent MVPA studies (Gallivan et al. 2013b; Chen et al. 2016, 2018; Turella et al. 2020; Monaco et al. 2020).

The second aim of the study was the description of the interplay between cortical regions of the ventral stream and of parieto-frontal pathways within the tool network. Our MVPA results can provide indirect evidence about the possible functional interactions underlying pantomimed movements by comparing how action and goal-related information is differently represented during the two phases of the task (planning and execution). Nevertheless, MVPA can only partially describe the functional interactions between regions of the tool network, as this type of evidence is not sufficient to establish the direction of information exchange within the network.

To directly test the direction of the interactions between the ventral stream and of parieto-frontal motor pathways, we focused our analysis only on one of the two tasks of our study: performing pantomime of tool use (i.e. grasp-to-use condition). This task has been widely adopted in the clinical practice for evaluating patients with apraxia (Goldenberg 2017). Moreover, lesion-mapping studies adopting univariate (Buxbaum et al. 2014; Hoeren et al. 2014; Weiss et al. 2016) and multivariate approach (Sperber et al. 2019) indicated three cortical sites causally associated with impairments in tool use pantomime: the posterior temporal cortex, the IPL and inferior frontal cortex. However, the functional interplay and the direction of the cortical interactions between these brain areas—which allows to successfully orchestrate this task in the healthy brain—are still largely unexplored.

To this aim, we investigated the direction of information flow between these three cortical areas adopting a type of connectivity analysis—i.e. dynamic causal modelling (DCM)—which allows to describe the type and direction of communication between brain regions. Our prediction would be that temporal and parieto-frontal nodes of the tool network should show an interplay during this task, but the exact pattern of functional interactions is difficult to predict. The description of the nature of this interplay would provide a crucial element to understand the role of these three cortical regions in the performance of pantomimed movements.

## Materials and methods

### Participants

Seventeen right-handed participants (seven females, mean age 28.35, age range 24–44 years) took part in the experiment. All participants gave written informed consent for their participation in the study and were reimbursed for their time. The ethical committee for human research of the University of Trento approved the protocol of the study which was prepared in accordance with the Declaration of Helsinki.

### Experimental task and design

Participants were requested to perform a delayed motor task within the MR scanner which consisted in executing a specific pantomimed movement following a “go” cue delivered after a fixed time interval. Pantomimes did not involve any interaction with a real tool and were executed moving the forearm and hand only, without the involvement of the arm and the shoulder. Participants were cued to pretend to use two different tools for the pantomimes, either scissors or an axe. The movement included an initial grasping component in which the participant pretended to grasp the cued tool. This initial grasping action was followed either by a pantomime of the use of the tool (‘grasp-to-use’ condition) or by a pantomime consisting in moving the tool laterally (‘grasp-to-move’ condition). Instructions were delivered through auditory cues.

These experimental conditions were embedded in a 2 × 2 factorial design, including as experimental factors: (a) the type of pantomimed action (either ‘grasp-to-move’ or ‘grasp-to-use’) and (b) the adopted tool (either scissors or axe). We selected these two “virtual” tools as we can exploit their functional properties to characterize action and goal encoding with MVPA. The two tools differ in terms of the kinematics of their associated pantomimes, but they can be used to achieve the same final goals, as they can be moved or used to cut.

### Experimental session and trial

Each experimental session consisted of eight functional runs (duration 7 min each). After an initial baseline period (duration 16 s), each run included 16 trials (four repetitions for every condition). After the last trial ended, an additional baseline period was presented (duration 20 s).

**Fig. 1:**
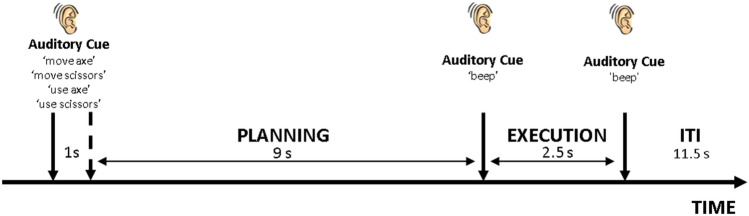
Timeline of the experimental trial. The trial started with a verbal cue instructing the subject about the type of action to pantomime (duration 1 s). After 9 s of delay (planning), the subject was instructed with an auditory cue (‘beep’) to perform the pantomime (execution) with the right dominant hand. After 2.5 s, another auditory cue (‘beep’) indicated the end of the trial. The participant waited for a new cue to start the following trial (inter-trial interval, ITI, duration 11.5 s)

Each experimental trial followed the same structure (Fig. 1, for a similar paradigm see Monaco et al. 2019, 2020). We delivered a verbal cue (duration 1 s) to the participants signaling the action to be performed. The verbal cues corresponded to the four experimental conditions (i.e. ‘use scissors’, ‘move scissors’, ‘use axe’ or ‘move axe’). After the verbal cue, the participant had to wait for 9 s (planning phase) until an auditory signal indicated to execute the planned action. Participants had to perform the instructed pantomime with the right dominant hand (execution phase). Another auditory instruction (‘beep’) signaled to return to the starting position. A baseline period (11.5 s) was presented between trials (inter-trial interval, ITI).

Participants performed the task with the head in a “standard position” within the MR coil. Throughout the entire experimental session, they had to look at a fixation cross projected on a screen through a mirror placed on the head coil. The right hand was kept at rest on an MR-compatible button box fastened on their chest with a Velcro belt. The button box allowed the recording of the reaction times (RTs) during the MR session. Stimulus delivery and response collection were controlled with the Presentation software (version 16, Neurobehavioural Systems, https://www.neurobs.com/).

Before the MR session, participants were trained to perform the pantomimes correctly. The experimenter explained how to pantomime the grasp-to-use and grasp-to-move actions for both tools. They were requested to perform the movement as if they were using the real tool. The participants practiced the task outside the MR scanner under the supervision of the experimenter to ensure the understanding of the timings and of the to-be-performed pantomimes.

Within the MR room, participants were also trained to perform the pantomime without moving the upper arm and the shoulder while lying on the scanner bed. We asked them to pretend to grasp the object from their abdomen and to perform the pantomime without excessive emphasis to avoid abrupt movements within the MR scanner. In addition to this initial training, the fMRI session was also recorded with an MR-compatible video camera, and the performance of the participants checked offline for possible errors (e.g. performed the wrong pantomime).

### MR data acquisition

The parameters for data acquisition were similar to previous published work of our lab (Monaco et al. 2019, 2020; Turella et al. 2020). All the MR data were acquired with a 4 T Bruker MedSpec scanner using an 8-channel head coil. T1-weighted anatomical scan (MP-RAGE, 176 axial slices, 1 mm isotropic voxels) images were acquired at the beginning of every session for each participant. The BOLD functional images were acquired with a T2^*^ echo-planar imaging (EPI) sequence. Before each functional run, we collected the point-spread function (PSF) of the subsequently acquired sequence to correct for possible distortions (Zaitsev et al. 2004). We acquired 28 slices tilted to be parallel with the ACPC line (TR 2 s, TE 33 ms, FOV 64 × 64 mm, in-plane resolution 3 × 3, slice thickness 3 mm, gap size 0.45 mm). For the main experiment, participants completed eight runs of 210 volumes each (duration 7 min). After the last run, we collected functional data for a “tool” localizer session for each subject (116 volumes, duration 3 min and 52 s, same acquisition parameters as in the runs of the main experiment).

### Tool localizer session

After the main experimental session, participants underwent a functional localizer to identify the tool network (Gallivan et al. 2013c, a). A single functional run was collected for each participant. The localizer consisted of alternating blocks (duration: 16 s each) presenting 18 images of tools or 18 images of scrambled tools (6 blocks per condition). A blank image with a fixation dot was displayed at the beginning and the end of the functional localizer (duration 20 s each). Participants had to perform a one-back task, pressing a button when the same tool or scrambled image was presented consecutively.

### Behavioural analysis

Offline analysis of the video-recorded fMRI sessions showed that participants could perform the task with a high level of accuracy. We removed trials in which participants: (a) did not perform the correct pantomime, (b) did not perform any action and/or (c) released the button before the auditory signal. These trials were considered errors and were removed in the following behavioural and fMRI analyses. The total number of errors was eight. Six participants performed one error, and a single participant performed two errors.

We extracted RTs defined as the time interval between the first auditory cue (“beep”) and the time when participants lifted their hand from the button box to perform the pantomime. RTs above two standard deviations from the mean were considered as outliers and removed. Then, a repeated measure ANOVA (factors tool, action type) was performed on the RTs. RTs were collected for sixteen subjects. Due to technical problems with the button box, responses for one participant were not registered.

### MR data pre-processing

Data pre-processing and analysis were performed with MatLab (MathWorks) and the SPM12 toolbox (http://www.fil.ion.ucl.ac.uk/spm/software/spm12/). We discarded the first five volumes of each run to avoid the saturation effect. We realigned and applied slice-timing correction to the functional data. Then, the T1-weighted anatomical image was co-registered with the realigned functional mean EPI image. Normalization of the anatomical image was performed adopting the unified segmentation approach implemented in SPM12. The resulting normalization parameters were applied to the functional images (resampling voxel size at 3 × 3 × 3 mm). Spatial smoothing was applied to functional data (8 mm FWHM Gaussian kernel) only for the univariate analysis. A high-pass filter (128 s) was also applied to the time series.

### Tool localizer: univariate analysis

For each participant, a general linear model (GLM) was estimated for the tool localizer. The predictors of interest consisted of the two categories of the presented images: tools and scrambled tools. The predictors were created with boxcar functions convolved with hemodynamic response function (HRF). The duration of the boxcar function was equivalent to the duration of the block (16 s). In addition, we modelled movement parameters (3 rotations and 3 translations) as predictors of non-interest. As we were interested in localizing activity related to tool observation, we computed the *t* contrast at the group level between the two conditions of interest: tools vs scrambled tools. We adopted a cluster-forming threshold of *p* < 0.001 uncorrected at the voxel level and then adopted family wise error rate at the cluster level (*p* < 0.05 FWE-corrected) to control for multiple comparisons. The resulting activation map was used to independently select the regions of interest (ROIs) within the tool network.

### Selection of ROIs for MVPA

The first objective of the study was to characterize action and goal representations within the tool network during pantomimed movements. To this aim, we selected eight ROIs in the left hemisphere, based on previous investigations on hand (for review see Culham and Valyear 2006; Culham et al. 2006; Vesia and Crawford 2012; Turella and Lingnau 2014; Gallivan and Culham 2015) and tool movements (for review see Johnson–Frey 2004; Lewis 2006; Valyear et al. 2017). These ROIs were similar to the ones selected in a fMRI study on hand and real tool movements (Gallivan et al. 2013c) which allows an indirect comparison of results across the two investigations.

We adopted the tool localizer to identify the ROIs independently from the main experiment (see the previous section). The selected ROIs within the tool network included:

- the left dorsal premotor cortex (PMd), at the junction between the precentral sulcus and the superior frontal sulcus (Valyear et al. 2012; Gallivan et al. 2013c),
- the left ventral premotor cortex (PMv), located within the precentral gyrus (Gallivan et al. 2011, 2013c; Valyear et al. 2012),
- the left superior parietal lobule (SPL), located posteriorly to the postcentral sulcus and superiorly to the intraparietal sulcus (Lewis 2006),
- the left superior-parieto-occipital cortex (SPOC) in the superior end of the parieto-occipital sulcus (Vesia et al. 2010; Gallivan et al. 2013c),
- the left anterior intraparietal sulcus (aIPS) located in the junction between intraparietal sulcus and postcentral sulcus (Culham et al. 2003; Grefkes and Fink 2005; Valyear et al. 2007; Valyear and Culham 2010; Gallivan et al. 2013c),
- the left supramarginal gyrus (SMG), lateral to the segment of IPS and posterior to the lateral sulcus (Lewis 2006; Gallivan et al. 2013c),
- the left posterior middle temporal gyrus (pMTG) within the posterior portion of the ventral stream (Gallivan et al. 2013c),
- the left primary motor area (M1), identified in the ‘hand knob’ along the anterior part of the central sulcus (Yousry 1997), has been localized adopting a univariate contrast from the main experiment (execution vs baseline, see below).

For each ROI, we started from the activation at the group level obtained from univariate analysis of the tool localizer. Within a radius of 8 mm from the group peaks, we created subject-specific ROIs centered on the peaks extracted from the activation map of the same contrast for each participant. ROIs were created as spheres with a radius of 12 mm centered on this subject-specific peaks. Table 1 contains the coordinates of the peaks at group level together with subject-specific peaks and their standard deviations.

**Table 1:** ROIs considered for MVPA

ROI	Group level peak coordinates			Mean single subject coordinates (± standard deviation)		
	*X*	*y*	*Z*	*X*	*y*	*Z*
Left pMTG	−51	−55	5	−49 ± 3	−55.4 ± 4.2	5.6 ± 4.1
Left SMG	−57	−38	34	−50.5 ± 4	−25.4 ± 3.4	36.9 ± 4.4
Left PMv	−42	2	44	−43.3 ± 3.2	2 ± 3.9	42.1 ± 3.9
Left aIPS	−39	−37	44	−39.3 ± 4.4	−36 ± 4.1	43.6 ± 4.1
Left PMd	−27	−7	62	−28 ± 4.1	−4.1 ± 5.2	57.5 ± 3.6
Left SPOC	−24	−76	29	−25.4 ± 2.9	−75.1 ± 4.4	30.1 ± 4.2
Left SPL	−30	−52	56	−29 ± 3.1	−50.9 ± 3	55.6 ± 5
Left M1	−36	−28	59	−33.8 ± 2.9	−24.8 ± 2.2	58 ± 3.9

The table reports the coordinates for each ROI at group level, the mean of the coordinates extracted from all the subjects, and the standard deviation for the three coordinates (*x*, *y*, *z*) of each ROI. The group peak coordinates were extracted from the independent localizer (observing images of tools vs. scrambled) for all the ROIs, expect M1. For M1, we identified the group peak with a different contrast (execution vs. baseline). Coordinates are reported in MNI space

### MVPA

For MVPA, we defined a GLM considering the three parts of the task separately: the cue, the planning and the execution phase (Fig. 1). A similar approach has been adopted in recent investigations on the neural correlates of hand movements (Chen et al. 2014; Cappadocia et al. 2016; Monaco et al. 2019).

For each participant, we estimated a GLM on unsmoothed data modelling every single trial for each experimental condition. A total of 384 regressors of interest were considered, originating from the 4 experimental conditions (move axe, move scissors, use axe, use scissors) × 3 phases (cue, planning, execution) × 4 repetitions per run × 8 runs. In addition, we modelled movement parameters (3 rotations and 3 translations), and errors, if present, as predictors of no interest.

COSMoMVPA toolbox (Oosterhof et al. 2016, http://www.cosmomvpa.org/) was adopted to perform MVPA analysis. We performed a ROI-based MVPA analysis (Kriegeskorte and Bandettini 2007) adopting Linear Discriminant Analysis (LDA) as classifier. We considered subject-specific ROIs defined with the independent tool localizer (see the previous section).

**Fig. 2:**
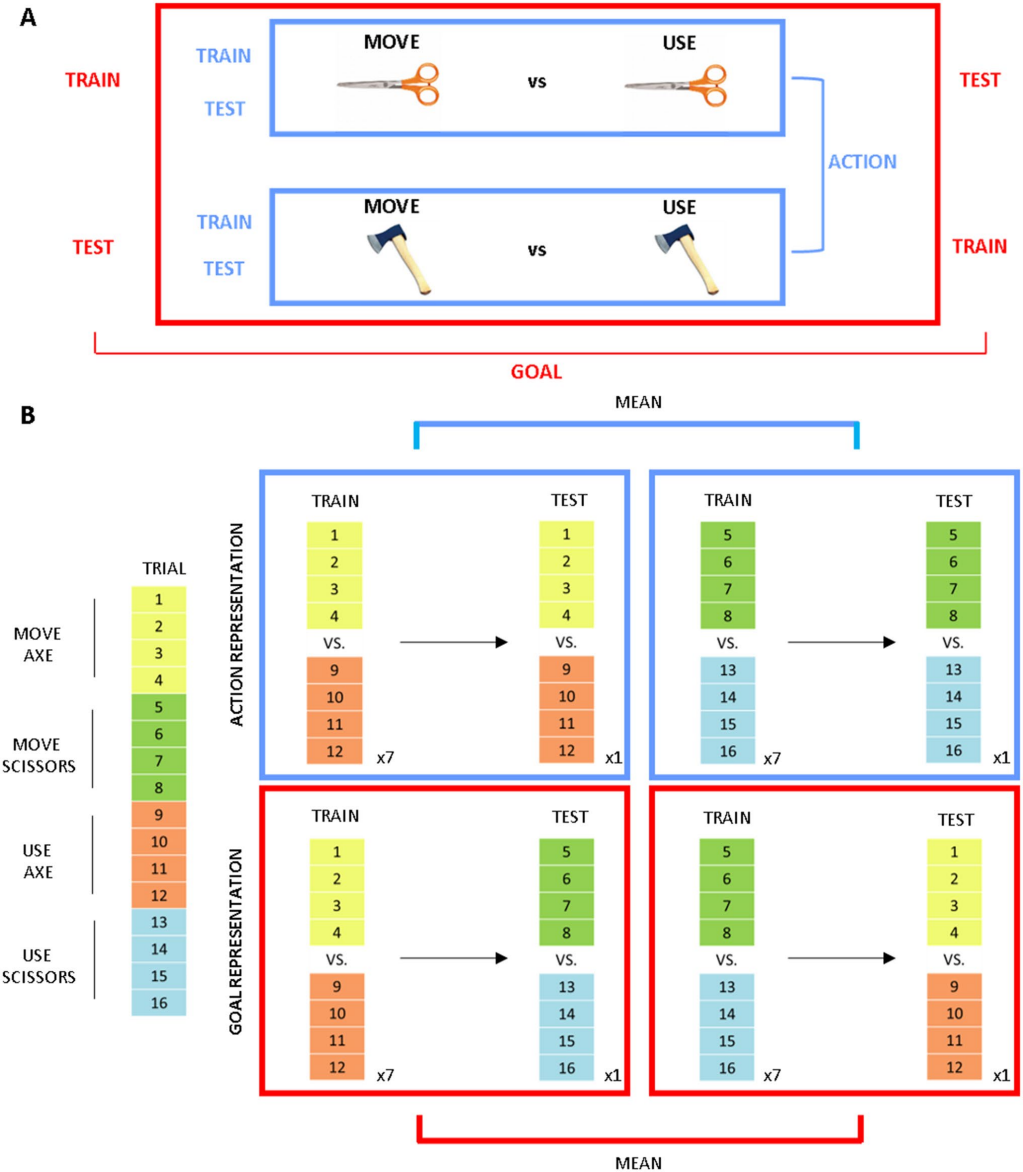
**A** MVPA pairwise comparisons. To test for action encoding (blue box), we performed an MVPA analysis, comparing trials for the grasp-to-move and grasp-to-use conditions for a specific tool. To test for goal encoding (red box), we adopted cross-decoding. We trained the classifier on the pairwise comparison between grasp-to-move vs grasp-to-use for one tool (e.g. axe) and then tested the classifier for the same comparison on the other tool (e.g. scissors). Then, we performed the same analysis but switching the trials adopted for training (e.g. scissors) and testing (e.g. axe) the classifier (**B**). Cross-validation approach. Each experimental run comprised 16 trials, 4 for each experimental condition (left part of the panel). Decoding accuracy was estimated with a leave-one-run-out cross-validation approach. We trained the classifier on single trials of seven runs (4 trials × 7 runs per condition) and then testing the classifier on the trials of the remaining run (4 trials × condition). For action representation (blue box in the upper part of p anel [**B**]), we trained the classifier on a specific pairwise comparison (e.g. grasp-to-use scissors vs. grasp-to-move scissors) and tested the classifier on the same pairwise comparison. We did this procedure separately for the two tools and then averaged the decoding accuracy values for these two comparisons. For goal representation (red box in the upper part of panel [**B**]), we trained the classifier on one pairwise comparison (e.g. grasp-to-move vs grasp-to-use with the axe) and then tested the classifier on the same comparison on the other tool (e.g. scissors). Then, we performed the same analysis but switching the trials adopted for training (e.g. scissors) and testing (e.g. axe) the classifier. Finally, we averaged the decoding accuracy values for these two decoding combinations

For each ROIs, we tested two pairwise comparisons separately for intended (planning phase) and performed movements (execution phase). The aim was to investigate the encoding of (1) action information, representing specific pantomimes performed with the same tool (see blue box in Fig. 2A), and (2) goal information, representing the same final goal (grasp-to-move vs grasp-to-use) irrespective of the adopted tool (see red box in Fig. 2A).

Decoding accuracy was estimated with a leave-one-run-out cross-validation approach (summarised in Fig. 2B). For each participant, the classifier was trained on single trials of seven runs (4 trials per condition × 7 runs) and then tested on the trials of the remaining run (4 trials per condition), considering all the possible combinations. We excluded the error trials from the analysis by randomly selecting the same number of trials for each condition. This procedure ensured having the same number of trials for each condition when training and testing the classifier.

To test for action encoding, we trained and tested the classifier on data for the two different actions (grasp-to-move vs grasp-to-use), separately for each tool (scissors and axe); then, we calculated the average of these two decoding accuracy maps in every voxel (see blue box in Fig. 2A, B). This subject-specific average decoding accuracy was then tested at the group level.

As in previous neuroimaging studies (Gallivan et al. 2013b, c; Tucciarelli et al. 2015; Turella et al. 2016, 2020), we adopted cross-decoding to test for goal encoding, i.e. the representation of the final aim of the movement irrespective of the adopted tool. Here, we trained the classifier on the pairwise comparison between pantomimes for one tool and then tested the classifier on the same comparison but for the other tool (see red box in Fig. 2A, B). This procedure was performed in both directions. Then, we calculated the average of the two decoding maps.

For all the comparisons, we tested decoding accuracy at the group level against chance (50%) with a one-sample *t* test (one-tailed) and corrected for multiple comparisons (across all ROIs and comparisons) applying a false discovery rate correction (FDR, *q* < 0.05, Benjamini and Yekutieli 2001).

### Connectivity analysis: rationale

The second aim of the study was to characterize the connectivity profiles underlying pantomimed movements. For this analysis, we focused only on one of our task—i.e. grasp-to-use conditions—as it has been widely adopted in the clinical practice for evaluating apraxic deficits (Goldenberg 2017).

**Fig. 3:**
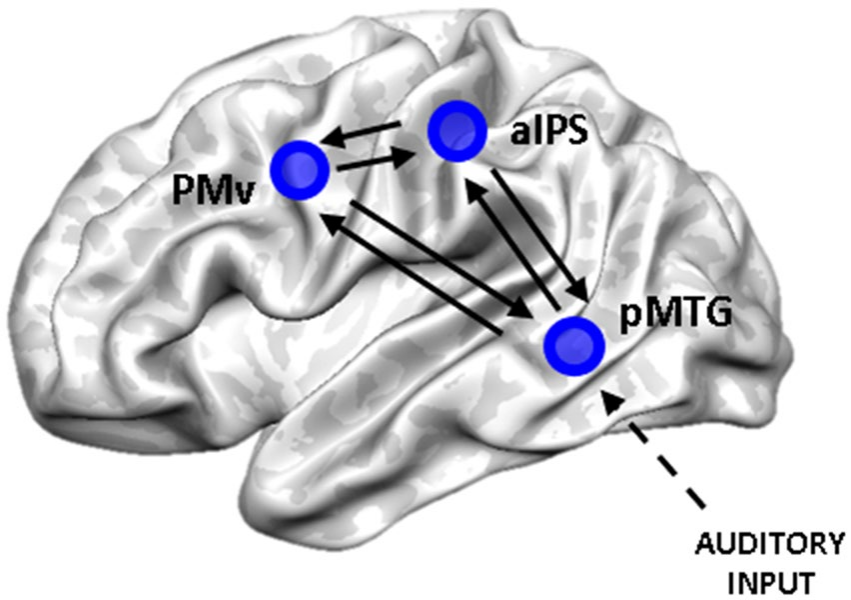
Connectivity analysis: ROIs position and connections. Visual representation of the three ROIs considered for connectivity analysis: aIPS, PMv and pMTG. The black arrows represent the intrinsic (bidirectional) connections between the nodes considered in the DCM analysis. We assumed that information (auditory input) enters the system from pMTG

We focused on three ROIs within the tool network: aIPS, pMTG, PMv (see Fig. 3). Previous studies showed the dorsolateral areas PMv and aIPS to be involved in tool and hand movements (Lewis 2006; Gallivan et al. 2013c). Univariate analysis showed that aIPS was activated both for real and pantomimed tool actions and it is the only brain region more strongly recruited during pantomime execution with respect to real tool movements (Hermsdörfer et al. 2007). Within the ventral stream, pMTG is well known to play a pivotal role in processing semantic knowledge regarding tools and tool-related actions (Johnson–Frey 2004; Lewis 2006; Binkofski and Buxbaum 2013; Lingnau and Downing 2015). The role of these three regions and their cortical location make them suitable nodes to investigate the functional interactions between the dorsolateral pathway and the ventral stream.

The aim of connectivity analysis was to examine: (1) how information was transferred between the temporal, parietal and frontal nodes of the tool network and (2) which was the direction of this interplay. To test the interactions between these two pathways, we performed a DCM analysis.

DCM has been shown to be suitable for understanding functional interactions during motor tasks in the healthy subjects and patients (Grefkes and Fink 2014), but also for investigating semantic information and manipulation knowledge about tools (Kleineberg et al. 2018). DCM approach requires to consider the simplest models to test the experimental question, in terms of number of regions and of possible reciprocal connections (Stephan et al. 2010). For this reason, we selected these three regions as they allowed defining the most economic and plausible anatomical model to test the functional interactions between these two functional pathways. DCM uses univariate signal changes over time to describe the coupling between the considered areas (intrinsic connectivity) and how a specific experimental condition or task can affect the functional communication between these nodes (modulatory effect).

### Connectivity analysis: DCM implementation

We adopted a specific analysis of the functional data for DCM. The functional data underwent the standard pre-processing analysis including realignment, slice-timing, normalization and smoothing (see MR data pre-processing Section). For model estimation, we considered the data of all the functional runs as if they were collected in single fMRI session, merging them into a single “concatenated” dataset (see https://en.wikibooks.org/wiki/SPM/Concatenation).

In our connectivity analysis, we considered only the execution phase of the task and focused on the “grasp-to-use” task (see previous sections). We identified subject-specific ROI starting from the peaks extracted from the localizer (Table 2). The center of each ROI was selected as the local maxima for the contrast (execution vs baseline) adopting the “concatenated” model estimated for each subject. Time series were extracted from these three ROIs, as the first eigenvariate within a sphere (radius 12 mm) around the individual maxima (see Table 2).

**Table 2:** ROIs adopted in the DCM analysis

ROIs name	Group level peak coordinates			Mean single subject coordinates		
	*X*	*y*	*z*	*X*	*y*	*z*
Left pMTG	−54	−46	8	−51.03 ± 3.05	−54.92 ± 4.23	5.17 ± 4.38
Left aIPS	−42	−40	47	−39.9 ± 3.03	−36.35 ± 3.36	48.21 ± 4.11
Left PMv	−48	5	44	−48.31 ± 4.16	4.82 ± 3.49	41.05 ± 3.87

The group level peaks were identified from the univariate contrast (execution vs. baseline) of the model estimated for DCM. We considered a sphere (radius 8 mm) with the center in the group coordinates. Single subject peak coordinates were extracted from the univariate GLM (execution vs. baseline) within this sphere. A ROI (radius 12 mm) was then created with the center in the individual peak. Peak positions are reported in MNI space

Based on the anatomo-functional constraints described in the previous sections, we considered the intrinsic connectivity—including forward and backward connections—between all these three regions (Fig. 3). Anatomical connections between aIPS and PMv should be direct (Davare et al. 2011; Vry et al. 2015), whereas connections between the ventral stream and PMv should be mediated through the IFG (Vry et al. 2015). It is not clear if there is a direct anatomical connection between pMTG and aIPS, but functional connectivity studies showed a strong coupling of pMTG with aIPS (and PMv) within the left hemisphere (Bracci et al. 2012; Hutchison et al. 2014). Moreover, lesion within the aIPS specifically modulated tool processing within the pMTG and the ventral temporal cortex (Garcea et al. 2019). Finally, we assumed that the auditory instructions of the task perturbed the activity of the considered network in pMTG (Fig. 3).

We aimed at understanding how the execution of our delayed-tool pantomiming task affected the connectivity profiles between the three selected regions. To this aim, we investigated the modulatory effect of the ‘grasp-to-use’ task on the functional coupling between these cortical areas.

**Fig. 4:**
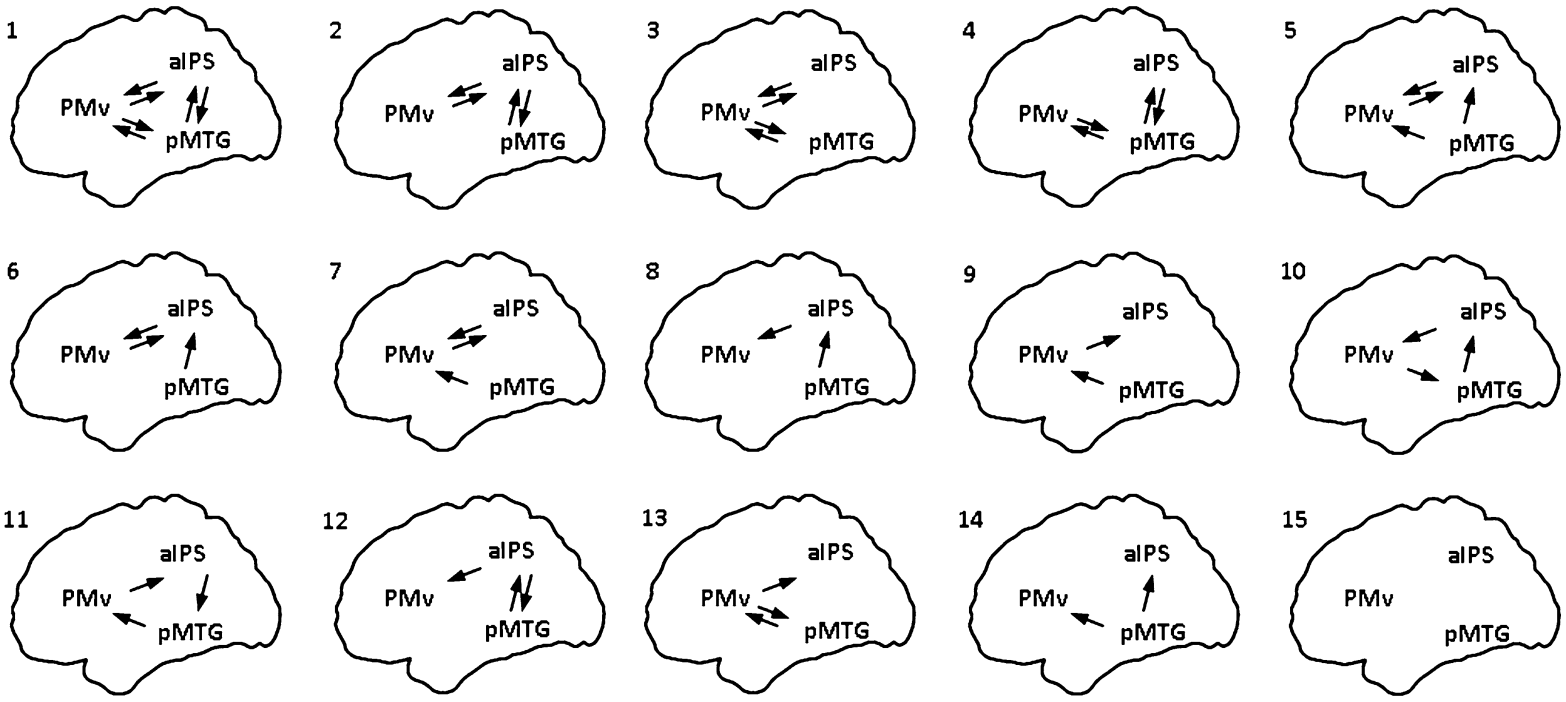
Models tested for the modulatory effects. We tested the modulatory effect of the ‘grasp-to-use’ task considering all the possible meaningful combinations of forward and backward modulatory connections between the considered nodes. A total of fifteen models were tested. The connections considered in each model are schematically represented in the image. To allow a direct comparison, we adopted the same numbers for the models in Table 3

We defined fifteen different models considering all the possible patterns of modulatory effect on the considered connections (see Fig. 4). We included models considering an interplay between the ventral stream and the dorsolateral pathway, as well as within the dorsolateral pathway, or a combination of the two. We adopted a random-effect Bayesian model selection to identify the model that better explains the given data in terms of the percentage of posterior probability compared to the other tested models (winning model). Then, we extracted for each subject the parameters of the winning model and averaged them across subjects and we statistically tested them at group level with a one-sample *t* test, adopting FDR correction for multiple comparisons (*q* < 0.05, Benjamini and Yekutieli 2001).

**Table 3:** Exceedance probabilities of the tested models

Single model	Exceedance probability
Model 1	0.7903
Model 2	0.0166
Model 3	0.0260
Model 4	0.0028
Model 5	0.0242
Model 6	0.0060
Model 7	0.0206
Model 8	0.0041
Model 9	0.0045
Model 10	0.0034
Model 11	0.0039
Model 12	0.0024
Model 13	0.0049
Model 14	0.0036
Model 15	0.0867

Adopting a BMS approach, between all the tested models, model 1 was the winning model (exceedance probability of 79%). The probabilities for all the models are listed in the table

## Results

### Behavioural results

We performed a 2 × 2 repeated measure ANOVA (factors tool and type of action) on the RTs of 16 participants. The main effect of tool (*F*_(1,15)_ = 0.905, *p* = 0.356), type of action (*F*_(1,15)_ = 1.155, *p* = 0.299) and the interaction between the two factors were not significant (*F*_(1,15)_ = 0.064, *p* = 0.804).

### ROIs selection with the tool localizer

The independent tool localizer allowed to identify the cortical regions recruited during the observation of tool (contrast: images of tools vs scrambled images). This contrast showed widespread recruitment within areas of the temporal lobe (comprising pMTG), of the parietal lobe (comprising SMG, aIPS, SPL, SPOC) and of the frontal cortex (comprising PMd and PMv).

**Fig. 5:**
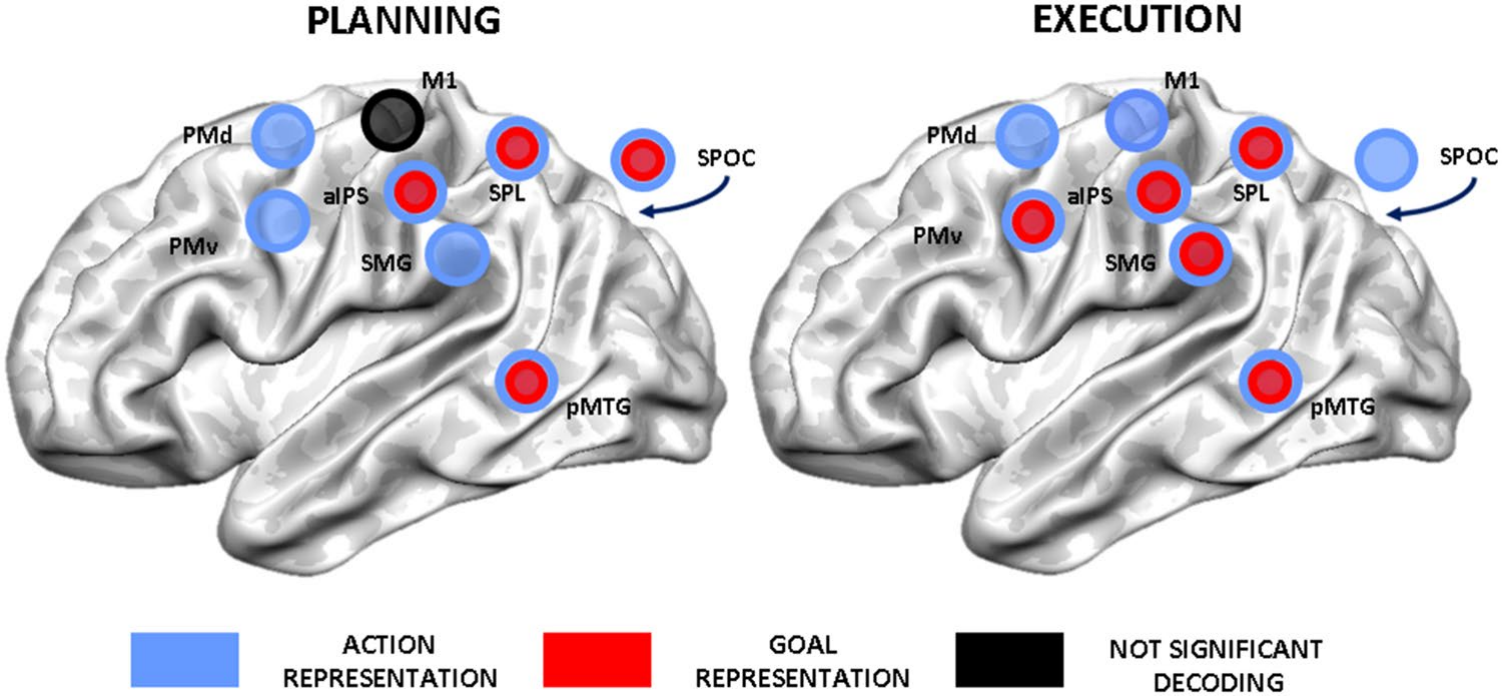
Summary of ROI-based MVPA results. This panel depicts a schematic representation of the results for the two phases of the task. We indicated ROIs with a significant decoding for action information with blue circles (FDR-corrected, *q* < 0.05) and for goal information with red circles (FDR-corrected, *q* < 0.05). A black circle indicates no significant decoding for the considered comparisons (FDR-corrected, *q* < 0.05)

Figure 5 shows the location of the selected ROIs within the tool network at the group level. We identified all the ROIs for MVPA, except for M1, using the tool localizer. The peak coordinates of M1 were extracted from the univariate contrast (execution vs. baseline) performed on the data of the main experiment.

### ROI-based MVPA results

**Fig. 6:**
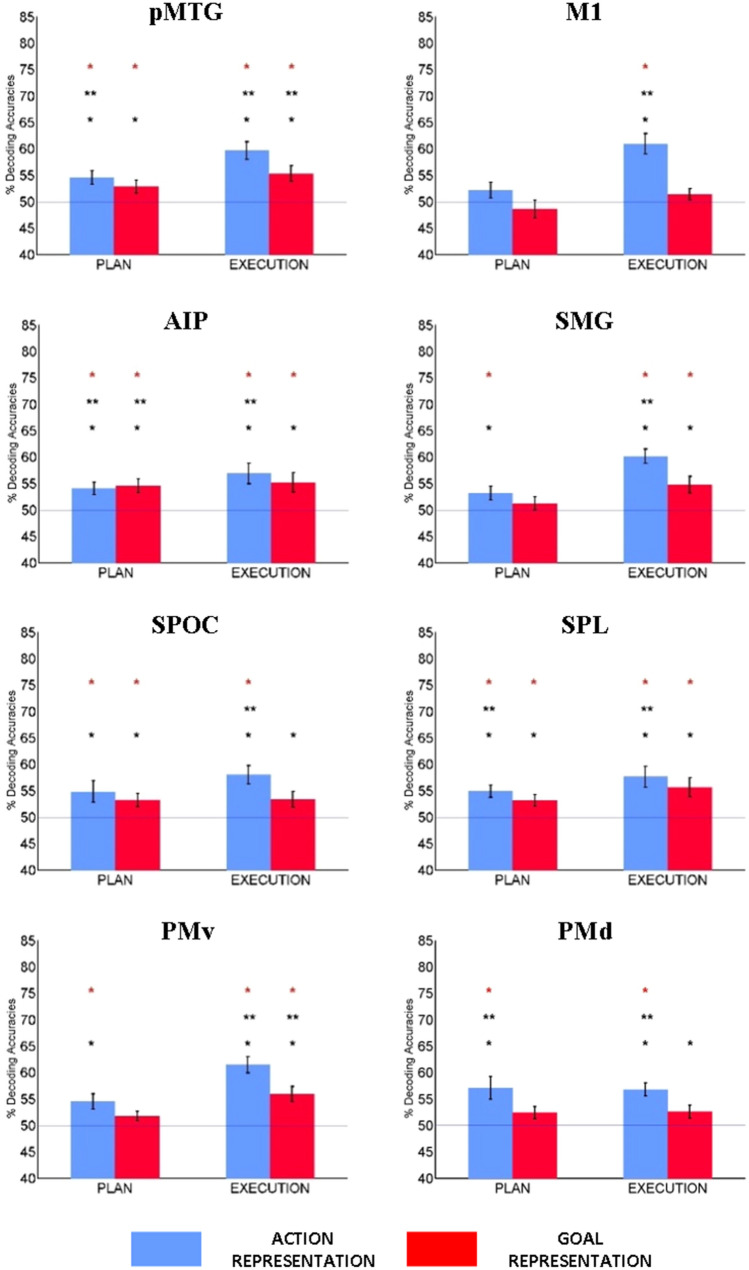
Decoding results for ROI-based MVPA. The bar graphs show the average decoding accuracy for ‘concrete’ action encoding (blue) and for ‘abstract’ goal encoding (red), separately for the planning phase (left) and in the execution phase (right). SPOC is located on the medial part of the brain, so it is represented outside the rendering of the template. Significant decoding is indicated with asterisks (*p* < 0.05*, *p* < 0.005**, FDR-corrected, *q* < 0.05, red star)

Our first aim was to describe action and goal encoding within the tool network happening during the two phases of our task. To this end, MVPA was performed separately for planning and execution. Figure 5 schematically summarizes the spatial patterns of significant decoding for the two phases (*q* < 0.05 FDR-corrected). For each pairwise comparison, we extracted decoding accuracy within each subject-specific ROI and we tested for significance at the group level (see Fig. 6).

ROI-based MVPA showed a different spatial distribution for decoding of action and goal-related information during the two phases of the task. During planning, decoding for the intention to perform a specific pantomime was significant in all the ROIs, except for M1 (Figs. 5 and 6). During execution, action information was represented in all the considered ROIs (Figs. 5 and 6).

A subset of the regions of the tool network represented both goal and action information. Preparatory information regarding the goal of the movement could be decoded in temporal (pMTG) and parietal regions (aIPS, SPL and SPOC) already during the planning phase of the task, whereas it was significant within PMv only during execution (Figs. 5 and 6).

Looking at MVPA results from another perspective provided insights also into the second aim of the study, i.e. characterizing the functional interactions behind pantomimed actions. During execution, we found a change in the encoding of goal information in two regions of the dorsolateral pathway (PMv and SMG), whereas pMTG, aIPS and SPL kept their representational content stable across the two phases of the task (see Figs. 5 and 6). This temporal difference in goal encoding between the planning and execution phase suggested that goal-related information seemed to reach frontal regions later compared to the posterior nodes of the network. These results suggested a possible transfer of information during pantomime execution between functionally connected areas of the tool network.

### DCM results

The second aim of the study was to investigate the connectivity profiles between the two main pathways involved in the execution of tool use pantomime: the dorsolateral pathway and ventral stream. Random-effect Bayesian model selection (BMS) showed that, out of fifteen tested models, the “winning” model was the one including all the forward and backward modulatory connections between the considered nodes (model one in Fig. 4). This model better explains our data with an exceedance probability of 79% (see Table 3).

**Fig. 7:**
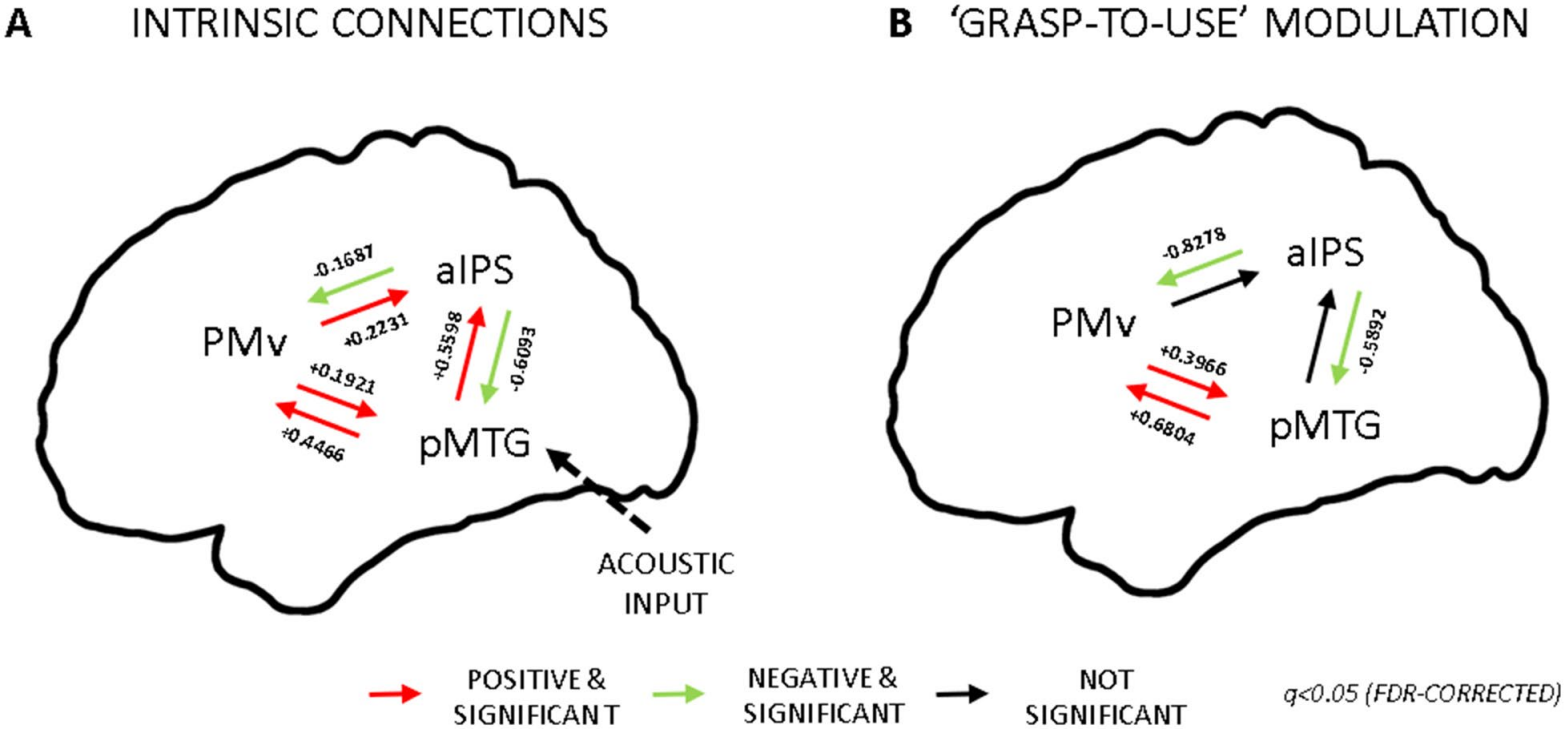
DCM Results. **A**. Intrinsic connectivity. The red arrows indicated positive coupling between two nodes, whereas the green arrows indicated negative between two nodes (*q* < 0.05 FDR-corrected). There was a bidirectional increased coupling between pMTG and PMv and unidirectional from pMTG to aIPS and from PMv to aIPS. Reduced communication was evident between aIPS and the other two functionally connected nodes **B**. Modulatory connectivity of the winning model. The ‘grasp-to-use’ task positively modulated the reciprocal connections between pMTG and PMV which showed enhanced coupling. The communication between aIPS and the other two nodes was instead reduced

Our DCM analysis showed the following average intrinsic effective connectivity patterns. All the intrinsic connections, both forward and backward, were significant (*q* < 0.05 FDR-corrected, summarised in Fig. 7). We found that the communication between pMTG and PMv during the execution of the task was enhanced in both directions. The same was true for the interplay between pMTG and aIPS and between PMv and aIPS. On the other hand, the functional coupling from aIPS to the other two nodes were significantly reduced during the execution of the task. These results showed a significant coupling between the regions of the ventral and the dorsolateral pathway.

Then, we examined directed modulatory effects, i.e. how the communication between the two considered pathways was modulated by the “grasp-to-use” task (modulatory effect). Four out of six connections were significant (*q* < 0.05 FDR-corrected, summarised in Fig. 7B). Overall, aIPS showed a decreased coupling with the other two nodes (pMTG, PMv), whereas there was enhanced bidirectional interplay between pMTG and PMv.

### Caveats for interpreting DCM analysis

We acknowledged the presence of possible factors which might have contributed to the specific pattern of connectivity shown in our study. First, the engagement of the ventral stream in our study might be caused by the specific selection of pantomime, which seemed to engage specifically the ventral pathway (Vry et al. 2015). Second, the adoption of a delayed pantomiming task might have also caused the recruitment of the ventral stream. It has been suggested that the ventral stream might be particularly involved in tasks requiring a delayed action and/or memory-based movements (Milner and Goodale 2006, 2008). Third, we cannot exclude that the directionality of the flow of information found with our connectivity analysis might be at least partially determined by the adoption of a non-visually guided task and by the delivery of the cue and go signals in the auditory modality. It is possible that a visually guided task might engage less the ventral stream. Nevertheless, a recent study adopting visually guided tool and hand actions showed functional interactions between temporal and fronto-parietal regions (Hutchison and Gallivan 2018).

Even considering these limitations, our analyses showed task-specific functional interactions within the ventral and the dorsolateral pathway, suggesting communication between these two pathways.

## Discussion

Our study aimed at providing novel insights on the neural correlates underlying pantomimed movements at two different levels. First, we wanted to characterize how action- and goal-related information are encoded within the tool network during the planning and execution of this type of movements. Second, we aimed at providing a description of the functional interactions between the ventral stream and parieto-frontal motor pathways happening during pantomimed movements.

With respect to the first aim, our ROI analysis showed a widespread encoding of action-related information within almost all the regions of the tool network. Significant decoding for goal information was present within the posterior parietal and temporal nodes of the network already during the planning phase, whereas it was present within frontal cortex only during the execution phase. Our findings showing the interplay between goal and action encoding in frontal and parietal regions are in line with previous human fMRI investigations (Gallivan et al. 2013b; Gallivan and Culham 2015; Turella et al. 2020) and also with electrophysiological evidence in non-human primates (Caminiti et al. 2017; Filippini et al. 2018; Hadjidimitrakis et al. 2019).

With respect to the second aim, the different spatial distribution of goal encoding during the two phases of the task suggested a possible exchange of information from the ventral stream and/or the parietal regions to the frontal cortex. In addition, our connectivity analysis showed an increased bidirectional coupling between pMTG and PMv during the execution of pantomime of tool use, suggesting a possible supportive role of ventral stream in orchestrating the normal performance of this type of movements. We will discuss further the implications of our findings in the next sections.

### Representation of action information during pantomimed movements

A recent MVPA investigation distinguished the functional selectivity of the regions within the tool network, by decoding specific movements (grasping vs. reaching) performed either with a real tool or only with the hand (Gallivan et al. 2013c). During planning, Gallivan et al. (2013c) showed that the intention to perform hand and tool movements are represented differently within the parieto-frontal networks and the ventral stream. Several regions within the network hosted overlapping but separate representations for planned hand and tool movements (M1, aIPS) or showed representations of shared goals across effectors (PMd, PMv, posterior IPS). The authors identified cortical areas representing only planned hand movements [i.e. SPOC, extrastriate body area (EBA)] from areas encoding just tool movements (i.e. pMTG, SMG)—suggesting a specialization of these last regions for real tool actions.

Our MVPA study complemented these results showing that—during planning—even specific exemplars of pantomimed movements were represented within similar regions of parieto-frontal networks (PMv, PMd, aIPS, SPL, SPOC). Moreover, upcoming pantomimes were also represented within areas of the tool network (SMG and pMTG) previously shown to be selective only for movements performed with real tool—even if our task did not involve interactions with real objects.

Of interest for the present study is the description of action encoding within the temporal lobe during movement planning. This is in line with the increasing number of evidence showing that the lateral occipito-temporal cortex (LOTC), comprising pMTG, represented specific motor features of upcoming actions, such as the adopted effector and the type of hand-object interaction (Lingnau and Downing 2015; Gallivan and Culham 2015). Following this interpretation, decoding of action information within pMTG might represent the specific parameters about upcoming (pantomimed) tool movements (Gallivan et al. 2013c) and/or a specific hand-tool relationships (Bracci et al. 2012; Bracci and Peelen 2013) which might be used to define a final desired state template to be achieved during action execution (Buxbaum 2017).

Overall, action-related encoding within the tool network suggested that pantomimes are a specific hand movement category which relies, at least partly, on the same cortical regions engaged during real tool actions by recruiting tool-selective regions of the temporal and parietal cortex.

### Representation of goal information during pantomimed movements

During the planning and the execution of pantomimes, our cross-decoding MVPA showed goal encoding within the temporal (pMTG) and parietal nodes (aIPS, SPL) of the tool network. Other regions (PMv, SMG) showed decoding of goal-related information only during the execution phase of the task. To be fully appreciated, our results need to be considered within a broader perspective to understand the possible role of temporal and parietal regions in pantomimed movements.

A general framework to interpret our results might come from a recent investigation on the simple observation of hand and tool images (Bracci et al. 2016). Bracci et al. (2016) showed that LOTC, possibly comprising also pMTG, hosted neural representations of action and object category, whereas the aIPS hosted only action information. The authors proposed that LOTC might integrate object and action information, representing a critical hub for linking more posterior temporal regions, involved only in perceptual processing, with parietal areas—involved in representing action information (Bracci et al. 2016).

Following this line of interpretation, decoding of goal information during action planning in pMTG might be related to the representation of the final goal to be obtained irrespective of the specific means and adopted tool. In parallel, the recruitment of the ventral stream during action preparation might also support the retrieval of semantic information about tools—i.e. their general function irrespective of the specific means and memory-based experiences about their typical use—which needs to be integrated with goal information into the to-be-performed pantomime (Lingnau and Downing 2015). Bidirectional exchange of task-relevant information between IPL (aIPS, SMG) and LOTC (pMTG) support the idea of an integrative role of LOTC (Almeida et al. 2013; Garcea et al. 2019). LOTC might be particularly relevant in planning pantomimes, as in this type of movement, the information about the tool is internally generated.

With respect to the parietal cortex, we showed encoding of goal information also within two regions of the IPL (aIPS and SMG) which might subtend different functional roles during pantomimes. Goal encoding in aIPS was evident during both planning and execution—in line with previous investigations (Gallivan et al. 2013b; Turella et al. 2020)—whereas SMG hosted goal-related information only during execution.

Recent fMRI studies adopting MVPA (Chen et al. 2016, 2018) reported the representation of similar goal-related information within IPL during the execution of pantomimes of tool movements. The first investigation (Chen et al. 2016) showed that tool-preferring regions of both parietal and temporal cortex encode goal-related information. As in our study, it was possible to decode goal information—invariant to the identity of the adopted tool—within IPL (aIPS and SMG) during pantomime execution. A further confirmation of goal encoding within IPL was evident in a subsequent study of the same group (Chen et al. 2018). Here, cross-decoding for goal-related information was possible within both SMG and aIPS during two different tasks: the execution of tool pantomime and the identification of visually presented tools. Nevertheless, only within SMG, decoding of goal information was independent from the performed task (Chen et al. 2018). This last result suggested a possible dissociation in the representational content hosted within aIPS and SMG.

Hints for a possible dissociation between the causal role of aIPS and SMG during pantomimed movements come from a recent lesion study (Watson and Buxbaum 2015). In this investigation, aIPS and pMTG were associated with a general impairment in the performance of tool pantomime (Watson and Buxbaum 2015), whereas SMG seemed to play a different role, more related to action selection (Buxbaum 2017). Indeed, lesions in SMG (and IFG) were associated with impairment in the selection of the to-be-performed pantomimed action (e.g. move vs. use) among possible candidates (Watson and Buxbaum 2015). Information from pMTG and aIPS might be the input to SMG, providing the possible candidates to be selected (Buxbaum 2017). This transfer of information might be supported by the temporal difference in encoding—from the planning to the execution phase—shown in our data, suggesting a transfer of goal information from pMTG and/or aIPS to SMG.

Significant decoding of goal information was also shown in superior parietal cortex, i.e. within SPL and SPOC. This information might be related to the maintenance of a representation of the intended general outcome to be subsequently performed (move vs. use) which might be adopted for subsequent online monitoring within both SPL and SPOC.

Still, SPOC showed a unique pattern of results, as it showed an opposite pattern with respect to other regions (SMG, PMv), shifting from representing action and goal information during planning to representing only action information during execution. This finding seems to support the central role of SPOC in the online control and guidance of an action and in the transformation of object-related information into a possible motor program (Gallivan et al. 2011; Vesia et al. 2017) in line with neurophysiological work on caudal parietal areas in monkeys (Filippini et al. 2018; Hadjidimitrakis et al. 2019).

Our results confirmed and extended the role of parietal (SMG, aIPS, SPL, SPOC) and of the ventral stream (pMTG) as crucial cortical hubs for processing goal information, further characterizing the different contribution of these regions during pantomimed movements.

### Functional interactions between ventral stream and parieto-frontal motor pathways during pantomimed movements

It is difficult to directly compare MVPA and DCM results, as these two methods capture different aspects of fMRI signal. Nevertheless, these methods allowed to describe the communication between ventral stream and parieto-frontal motor pathways from two different but complementary perspectives. Our MVPA results showed a possible transfer of abstract goal information from the ventral stream and/or the parietal cortex to PMv, whereas DCM showed a task-specific bidirectional functional interplay between the temporal and frontal cortex.

Overall, our findings are in line with recent investigations supported the possible exchange of information between ventral stream and parieto-frontal motor pathways during hand actions (van Polanen and Davare 2015; Milner 2017; Hutchison and Gallivan 2018). Nevertheless, the dynamics of this interplay are still poorly understood (Cloutman 2013). Our study contributed to better understand these interactions in two ways.

First, we showed differences in the spatial patterns of goal encoding during the planning and execution of pantomimed movements. Our results suggested a possible transfer of information from the posterior nodes of the network (pMTG, aIPS), where the encoding of goal information is stable through time, to the more anterior one (PMv). There are two possible, and not mutually exclusive, alternatives for information to reach the frontal cortex: in one case, information could be transferred from pMTG to aIPS and from there (or indirectly from SMG) to PMv, in the other case, information could be transferred from temporal regions—comprising pMTG—to the IFG and then to the premotor cortex (PMv).

Our findings showed that the representational content within the tool network changed flexibly according to the evolution of the movement, supporting a dynamic representation of goal-related information. Goal information might be transferred from posterior to anterior regions during the unfolding of the movement. This exchange of information might be possible also through the functional interactions between ventral stream and parieto-frontal motor networks.

Second, our connectivity analysis provided complementary evidence for an interaction between these pathways. In this case, we described a reciprocal exchange of information between the frontal, parietal and temporal nodes of the network occurring during the execution phase of the task (intrinsic connectivity). Looking at the specific effect of our grasp-to-use condition (modulatory effect), we showed that pantomiming the use of a tool selectively enhanced the functional coupling between the pMTG and the PMv in both directions. This interplay might subtend a continuous exchange of information between these two pathways. Communication in one direction (PMv > pMTG) could consist in comparing the actual state of the movement stored in premotor cortices with the planned end-state stored in temporal regions. The other interaction (pMTG > PMv) might represent a feedback providing information on the comparison between the action which is performed and the originally planned end-state.

The modulatory effect of the grasp-to-use task demonstrated enhanced communication between the ventral and the dorsolateral pathway, but mainly through the connections between temporal and frontal regions. Our results supported the indirect conclusions of a study (Vry et al. 2015) combining fMRI and tractography. Based on fMRI activation patterns, tractography identified a ventral pathway, connecting temporal cortex to frontal regions, as the main cortical route specific for object-directed pantomime. Moreover, our data are in line with a recent fMRI investigation (Garcea and Buxbaum 2019), even if the authors considered different nodes of the tool network and adopted a different connectivity measure. This study (Garcea and Buxbaum 2019) showed increased functional connectivity between parietal, temporal and frontal nodes of the network during the planning and execution of pantomime of tool use. Our results extended these findings providing a description of the nature of the bidirectional functional interplay between temporal and frontal regions during tool use pantomime.

Overall, if we look at our MVPA and DCM results from a general perspective, we provided evidence for the involvement of pMTG—and possibly of other LOTC regions—in the planning and execution of pantomimed movements, but it is still difficult to define their functional role in motor control. During planning, LOTC and the dorsal stream might exchange information about the properties of the to-be-grasped objects (e.g. its function), of the upcoming action and/or of its expected sensory consequences (Gallivan and Culham 2015).

During execution, information hosted within LOTC and exchanged with the parieto-frontal pathways might be different. A possibility could be that an efference copy of the executed action hosted within LOTC might be represented and adopted for online monitoring and possible corrections. A complementary interpretation might point towards the possible transfer of information related to the intention behind the pantomimed action, mediating the communicative side of pantomime (Goldenberg 2017; Finkel et al. 2018).

Irrespective of the possible interpretations, our data highlighted the pivotal role of temporo-frontal bidirectional interactions for the performance of meaningful pantomimed movements. Further studies are needed to better understand the interactions within the tool network underlying this type of movements, unveiling also the possible role of homologous regions within the right hemisphere, as hinted by a recent investigation (see Watson et al. 2019).

## Conclusion

Pantomimes are a unique movement category. Although they do not involve any real interaction with objects in the environment, they retain the capacity to convey complex information about our intentions. This characteristic makes them a privileged window to understand how action and goal information is represented in the human brain.

Following this line of thinking, our data provided novel insights on how pantomimes are represented within the tool network both in terms of neural encoding and of functional interactions. This network provides the neural structure sustaining a distributed representation of action and goal information which is maintained and flexibly transferred within the network throughout the evolution of a pantomime—from its planning to its execution. Moreover, the reciprocal interplay between the ventral and parieto-frontal motor networks seem to be at the basis of the successful performance of this peculiar type of movements.

## Data Availability

The code, the results of univariate analysis and of MVPA can be requested from the corresponding author. The raw functional and structural data of our study may be made available upon request after a confirmation from the ethical committee of our institution.

## Code availability

The code, the results of univariate analysis and of MVPA can be requested from the corresponding author. The raw functional and structural data of our study may be made available upon request after a confirmation from the ethical committee of our institution.

## References

[CR1] Almeida J, Fintzi AR, Mahon BZ (2013). Tool manipulation knowledge is retrieved by way of the ventral visual object processing pathway. Cortex.

[CR2] Benjamini J, Yekutieli D (2001). The control of the false discovery rate in multiple testing under dependency. Ann Stat.

[CR3] Binkofski F, Buxbaum LJ (2013). Two action systems in the human brain. Brain Lang.

[CR4] Bracci S, Cavina-Pratesi C, Connolly JD, Ietswaart M (2016). Representational content of occipitotemporal and parietal tool areas. Neuropsychologia.

[CR5] Bracci S, Cavina-Pratesi C, Ietswaart M (2012). Closely overlapping responses to tools and hands in left lateral occipitotemporal cortex. J Neurophysiol.

[CR6] Bracci S, Peelen MV (2013). Body and object effectors: the organization of object representations in high-level visual cortex reflects body-object interactions. J Neurosci.

[CR7] Brandi M-L, Wohlschläger A, Sorg C, Hermsdörfer J (2014). The neural correlates of planning and executing actual tool use. J Neurosci.

[CR8] Buxbaum LJ (2017). Learning, remembering, and predicting how to use tools: distributed neurocognitive mechanisms: comment on Osiurak and Badets (2016). Psychol Rev.

[CR9] Buxbaum LJ, Shapiro AD, Coslett HB (2014). Critical brain regions for tool-related and imitative actions: a componential analysis. Brain.

[CR10] Caminiti R, Borra E, Visco-Comandini F (2017). Computational architecture of the parieto frontal network underlying cognitive motor control in monkeys. eNeuro.

[CR11] Cappadocia DC, Monaco S, Chen Y (2016). Temporal evolution of target representation, movement direction planning, and reach execution in occipital–parietal–frontal cortex: an fMRI study. Cereb Cortex.

[CR12] Chao LL, Haxby JV, Martin A (1999). Attribute-based neural substrates in temporal cortex for perceiving and knowing about objects. Nat Neurosci.

[CR13] Chao LL, Martin A (2000). Representation of manipulable man-made objects in the dorsal stream. Neuroimage.

[CR14] Chen Q, Garcea FE, Jacobs R, a., Mahon BZ, (2018). Abstract representations of object-directed action in the left inferior parietal lobule. Cereb Cortex.

[CR15] Chen Q, Garcea FE, Mahon BZ (2016). The Representation of object-directed action and function knowledge in the human brain. Cereb Cortex.

[CR16] Chen Y, Monaco S, Byrne P (2014). Allocentric versus egocentric representation of remembered reach targets in human cortex. J Neurosci.

[CR17] Cloutman LL (2013). Interaction between dorsal and ventral processing streams: where, when and how?. Brain Lang.

[CR18] Culham JC, Cavina-Pratesi C, Singhal A (2006). The role of parietal cortex in visuomotor control: what have we learned from neuroimaging?. Neuropsychologia.

[CR19] Culham JC, Danckert SL, DeSouza JFX (2003). Visually guided grasping produces fMRI activation in dorsal but not ventral stream brain areas. Exp Brain Res.

[CR20] Culham JC, Valyear KF (2006). Human parietal cortex in action. Curr Opin Neurobiol.

[CR21] Davare M, Kraskov A, Rothwell JC, Lemon RN (2011). Interactions between areas of the cortical grasping network. Curr Opin Neurobiol.

[CR22] Filippini M, Breveglieri R, Hadjidimitrakis K (2018). Prediction of reach goals in depth and direction from the parietal cortex. Cell Rep.

[CR23] Finkel L, Hogrefe K, Frey SH (2018). NeuroImage : clinical it takes two to pantomime : communication meets motor cognition. NeuroImage Clin.

[CR24] Gallivan JP, Chapman CS, McLean DA (2013). Activity patterns in the category-selective occipitotemporal cortex predict upcoming motor actions. Eur J Neurosci.

[CR25] Gallivan JP, Culham JC (2015). Neural coding within human brain areas involved in actions. Curr Opin Neurobiol.

[CR26] Gallivan JP, McLean DA, Flanagan JR, Culham JC (2013). Where one hand meets the other: limb-specific and action-dependent movement plans decoded from preparatory signals in single human frontoparietal brain areas. J Neurosci.

[CR27] Gallivan JP, McLean DA, Valyear KF (2011). Decoding action intentions from preparatory brain activity in human parieto-frontal networks. J Neurosci.

[CR28] Gallivan JP, McLean DA, Valyear KF, Culham JC (2013). Decoding the neural mechanisms of human tool use. Elife.

[CR29] Garcea FE, Almeida J, Sims MH (2019). Domain-Specific diaschisis: lesions to parietal action areas modulate neural responses to tools in the ventral stream. Cereb Cortex.

[CR30] Garcea FE, Buxbaum LJ (2019). Gesturing tool use and tool transport actions modulates inferior parietal functional connectivity with the dorsal and ventral object processing pathways. Hum Brain Mapp Hbm.

[CR31] Goldenberg G (2017). Facets of Pantomime. J Int Neuropsychol Soc.

[CR32] Grefkes C, Fink GR (2005). The functional organization of the intraparietal sulcus in humans and monkeys. J Anat.

[CR33] Grefkes C, Fink GR (2014). Connectivity-based approaches in stroke and recovery of function. Lancet Neurol.

[CR34] Hadjidimitrakis K, Bakola S, Wong YT, Hagan MA (2019). Mixed spatial and movement representations in the primate posterior parietal cortex. Front Neural Circuits.

[CR35] Hermsdörfer J, Terlinden G, Mühlau M (2007). Neural representations of pantomimed and actual tool use: evidence from an event-related fMRI study. Neuroimage.

[CR36] Hoeren M, Kümmerer D, Bormann T (2014). Neural bases of imitation and pantomime in acute stroke patients: distinct streams for praxis. Brain.

[CR37] Hutchison RM, Culham JC, Everling S (2014). Distinct and distributed functional connectivity patterns across cortex reflect the domain-specific constraints of object, face, scene, body, and tool category-selective modules in the ventral visual pathway. Neuroimage.

[CR38] Hutchison RM, Gallivan JP (2018). Functional coupling between frontoparietal and occipitotemporal pathways during action and perception. Cortex.

[CR39] Johnson-Frey SH (2004). The neural bases of complex tool use in humans. Trends Cogn Sci.

[CR40] Johnson-Frey SH, Newman-Norlund R, Grafton ST (2005). A distributed left hemisphere network active during planning of everyday tool use skills. Cereb Cortex.

[CR41] Kleineberg NN, Dovern A, Binder E (2018). Action and semantic tool knowledge—effective connectivity in the underlying neural networks. Hum Brain Mapp.

[CR42] Kriegeskorte N, Bandettini P (2007). Combining the tools: activation and information-based fMRI analysis. Neuroimage.

[CR43] Króliczak G, Frey SH (2009). A common network in the left cerebral hemisphere represents planning of tool use pantomimes and familiar intransitive gestures at the hand-independent level. Cereb Cortex.

[CR44] Lewis JW (2006). Cortical networks related to human use of tools. Neurosci.

[CR45] Lingnau A, Downing PE (2015). The lateral occipitotemporal cortex in action. Trends Cogn Sci.

[CR46] Milner AD (2017). How do the two visual streams interact with each other?. Exp Brain Res.

[CR1000] Milner D, Goodale M (2006) The visual brain in action. Oxford University Press. 10.1093/acprof:oso/9780198524724.001.0001

[CR1001] Milner AD, Goodale MA (2008). Two visual systems re-viewed. Neuropsychologia.

[CR47] Monaco S, Malfatti G, Culham JC (2020). Decoding motor imagery and action planning in the early visual cortex: overlapping but distinct neural mechanisms. Neuroimage.

[CR48] Monaco S, Malfatti G, Zendron A (2019). Predictive coding of action intentions in dorsal and ventral visual stream is based on visual anticipations, memory-based information and motor preparation. Brain Struct Funct.

[CR49] Oosterhof NN, Connolly AC, Haxby JV (2016). CoSMoMVPA: multi-modal multivariate pattern analysis of neuroimaging data in Matlab/GNU octave. Front Neuroinform.

[CR50] Rumiati RI, Weiss PH, Shallice T (2004). Neural basis of pantomiming the use of visually presented objects. Neuroimage.

[CR51] Sperber C, Wiesen D, Goldenberg G, Karnath H-O (2019). A network underlying human higher-order motor control: Insights from machine learning-based lesion-behaviour mapping in apraxia of pantomime. Cortex.

[CR52] Stephan KE, Penny WD, Moran RJ (2010). Ten simple rules for dynamic causal modeling. Neuroimage.

[CR53] Styrkowiec PP, Nowik AM, Króliczak G (2019). The neural underpinnings of haptically guided functional grasping of tools: An fMRI study. Neuroimage.

[CR54] Tucciarelli R, Turella L, Oosterhof NN (2015). MEG multivariate analysis reveals early abstract action representations in the lateral occipitotemporal cortex. J Neurosci.

[CR55] Turella L, Lingnau A (2014). Neural correlates of grasping. Front Hum Neurosci.

[CR56] Turella L, Rumiati R, Lingnau A (2020). Hierarchical action encoding within the human brain. Cereb Cortex.

[CR57] Turella L, Tucciarelli R, Oosterhof NNN (2016). Beta band modulations underlie action representations for movement planning. Neuroimage.

[CR58] Valyear KF, Cavina-Pratesi C, Stiglick AJ, Culham JC (2007). Does tool-related fMRI activity within the intraparietal sulcus reflect the plan to grasp?. Neuroimage.

[CR59] Valyear KF, Culham JC (2010). Observing learned object-specific functional grasps preferentially activates the ventral stream. J Cogn Neurosci.

[CR60] Valyear KF, Fitzpatrick AM, McManus EF (2017). The Neuroscience of Human Tool Use.

[CR61] Valyear KF, Gallivan JP, McLean DA, Culham JC (2012). fMRI repetition suppression for familiar but not arbitrary actions with tools. J Neurosci.

[CR62] van Polanen V, Davare M (2015). Interactions between dorsal and ventral streams for controlling skilled grasp. Neuropsychologia.

[CR63] Vesia M, Barnett-Cowan M, Elahi B (2017). Human dorsomedial parieto-motor circuit specifies grasp during the planning of goal-directed hand actions. Cortex.

[CR64] Vesia M, Crawford JD (2012). Specialization of reach function in human posterior parietal cortex. Exp Brain Res.

[CR65] Vesia M, Prime SL, Yan X (2010). Specificity of human parietal saccade and reach regions during transcranial magnetic stimulation. J Neurosci.

[CR66] Vry M-S, Tritschler LC, Hamzei F (2015). The ventral fiber pathway for pantomime of object use. Neuroimage.

[CR67] Watson CE, Buxbaum LJ (2015). A distributed network critical for selecting among tool-directed actions. Cortex.

[CR68] Watson CE, Gotts SJ, Martin A, Buxbaum LJ (2019). Bilateral functional connectivity at rest predicts apraxic symptoms after left hemisphere stroke. NeuroImage Clin.

[CR69] Weiss PH, Ubben SD, Kaesberg S (2016). Where language meets meaningful action: a combined behavior and lesion analysis of aphasia and apraxia. Brain Struct Funct.

[CR70] Yousry T, Schmid UD, Alkadhi H (1997). Localization of the motor hand area to a knob on the precentral gyrus. A New Landmark. Brain.

[CR71] Zaitsev M, Hennig J, Speck O (2004). Point spread function mapping with parallel imaging techniques and high acceleration factors: Fast, robust, and flexible method for echo-planar imaging distortion correction. Magn Reson Med.

